# Factors influencing newly graduated registered nurses' voice behaviour: An interview study

**DOI:** 10.1111/jonm.13742

**Published:** 2022-08-01

**Authors:** Karin Kee, Demi de Jong

**Affiliations:** ^1^ Department of Organization Sciences Vrije Universiteit Amsterdam Amsterdam The Netherlands

**Keywords:** communication, new graduate nurses, nursing, registered nurses, speaking up, voice behaviour

## Abstract

**Aim:**

To gain insight into the factors that affect newly graduated registered nurses' voice behaviour.

**Background:**

Employees with little work experience may experience difficulties with speaking up. Given that a lack of voice can negatively affect the delivery of safe client care and lower nurses' job satisfaction, it is important to understand which factors facilitate and hinder newly graduated nurses' voice behaviour.

**Methods:**

A qualitative descriptive study was conducted using semi‐structured interviews with 17 newly graduated registered nurses working in inpatient hospital settings.

**Results:**

In total, seven factors emerged from our data, which were grouped in four overarching themes. Whether newly graduated nurses speak up depends on (1) their levels of self‐confidence, (2) whether they feel encouraged and welcome to speak up, (3) their relationship with the voice target and (4) the content of their voice message.

**Conclusion:**

Factors that affect newly graduated nurses' voice behaviour are multifaceted, but mostly centre around time spent in and relationships at the workplace.

**Implications for Nursing Management:**

Nurse managers and colleagues can build an environment that fosters newly graduated nurses' voice behaviour. Specifically, induction programmes, assigning mentors and offering additional training can support newly graduated nurses in developing voice behaviour.

## 1 INTRODUCTION

Nurses are central to the working of care organizations (Garon, 2012). Not only do nurses possess unique information about patients' health conditions; they often also have ideas for how their work unit may operate more efficiently (Kee et al., 2021). For this to be effective, it is essential that nurses engage in ‘voice behaviour’ (Alingh et al., 2019)—defined as the different ways and means through which employees speak up, attempt to have a say in and potentially influence matters that affect their work or lives (Morrison, 2014). By speaking up, nurses can ensure the delivery of safe and high‐quality care (Levine et al., 2020) and can exert influence regarding work matters (Kee et al., 2021). This, in turn, may result in feelings of empowerment and being in control (Both‐Nwabuwe, 2020).

Yet, nurses are often hesitant to engage in voice behaviour (Hanson et al., 2020). Three reasons have been named for this (Okuyama et al., 2014). First, nurses may perceive voice behaviour as ‘risky’ (Morrow et al., 2016). As voice inherently tries to change the status quo, it may not always be responded to positively by managers and colleagues. As a result, nurses may face—and fear—negative consequences, such as being disrespected and ignored. Second, nurses may experience a sense of powerlessness, and perceive their voice is not being listened to, nor acted upon (Garon, 2012; Todorova et al., 2014). Finally, nurses may have learned that voice is not a type of behaviour that is expected of them (Morrow et al., 2016). Consequently, nurses may be more inclined to remain silent than to speak up (Garon, 2012).

Scholars have also studied factors that facilitate or hinder nurses' voice behaviour (Okuyama et al., 2014). On the individual level, research has shown that nurses who experience high levels of job satisfaction and identification with their job and team feel a need to contribute. They may do so by sharing their ideas and perspectives (Morrison, 2014). At the team and organizational level, the behaviour demonstrated by employees higher in status, such as managers and physicians, affect nurses' voice behaviour (Alingh et al., 2019; Weiss et al., 2018). Research has shown that nurses become hesitant to speak up, when they perceive or have experienced that authority holders act dismissive or aggressive towards them (Todorova et al., 2014). In contrast, voice is enhanced when nurses are invited and encouraged to speak up (Weiss et al., 2018).

What characterizes existing studies on nurses' voice behaviour is that they mainly focus on nurses with several years of work experience (e.g. Alingh et al., 2019; Garon, 2012). Yet, especially employees with little work experience may be hesitant to speak up (Morrison, 2014), such as newly graduated registered nurses (NGRNs). NGRNs are oftentimes not fully familiar with their team and organization and are still gaining knowledge and practice experience (Lyman et al., 2020). Consequently, NGRNs may feel overwhelmed. Moreover, the well‐entrenched hierarchy most hospitals tend to be characterized by, may result in feelings of inferiority among NGRNs (Rush et al., 2019). These factors may hinder NGRNs' voice behaviour. This is of particular concern in light of the global nursing shortage, as communication issues, such as a lack of voice behaviour, have been shown to negatively affect nurses' job satisfaction and retention (Both‐Nwabuwe, 2020).

Although the importance of NGRNs' voice behaviour is clear, factors that affect NGRNs' voice behaviour are not well documented. The purpose of this study was therefore to fill this gap. The study's findings can help NGRNs better prepare for nursing practice and can serve as a reference for nurse managers to initiate interventions aimed at creating a voice‐enhancing work environment for NGRNs.

## 2 METHODOLOGY

### 2.1 Design

A qualitative descriptive study was conducted using semi‐structured interviews. The use of such a design is deemed appropriate when the study aims to develop a better understanding of a phenomenon, based on perspectives from those who have experienced it (Doyle et al., 2020). Given that the factors that affect NGRNs' voice behaviour are not well understood in the literature, a qualitative descriptive design was selected. Ethical approval was granted by the authors' University Ethics Review Committee.

### 2.2 Respondents

Respondents were considered to be eligible for this study if they worked (1) as a registered nurse, (2) in an inpatient hospital setting and (3) if they had graduated from a university of applied sciences within the 2 years prior to this study. Initial selection of participants was performed by convenience and snowball sampling (Bryman, 2012). The authors first approached three existing professional connections. During a phone call, one of the researchers explained the purpose and design of the study and invited the NGRN for an interview. Voluntary participation and confidentiality and anonymity were ensured. NGRNs were then asked if they were willing to participate and if they had any questions. After obtaining their approval, an interview appointment was scheduled. All respondents received an informed consent form by mail, which they were asked to read and sign. At the end of the interview, respondents were asked if they knew other NGRNs, and, if so, were asked if they could ask the latter if they would be willing to participate in this study. Upon agreement, contact details were shared with the researchers, who then approached the potential respondents in the same way as described above.

### 2.3 Data collection

Data were collected through semi‐structured interviews with NGRNs. The use of semi‐structured interviews allowed for a systematic exploration of respondents' views and experiences, as well as space for the researchers for follow‐up queries and for the respondents to highlight other relevant topics (Bryman, 2012). The topic list that guided the interviews was aimed to acquire information about NGRNs' experiences with exhibiting voice behaviour. We drew upon interview guides developed by Garon (2012) and Schwappach and Gehring (2014), who have studied facilitating and hindering conditions to registered nurses' voice behaviour. We made slight changes to the questions, so as to ensure that they fit our research context and population. Specifically, we asked respondents about prior experiences with speaking up and what they considered to be facilitating and inhibiting conditions to their voice behaviour. We asked open‐ended questions, so as to grasp respondents' lived experiences and opinions (Bryman, 2012).

In total, 15 interviews were conducted with 17 respondents. Participant characteristics can be found in Table 1. Thirteen interviews were conducted by the second author in February 2021. During two interviews, two NGRNS were interviewed at the same time. Data saturation was reached after 13 interviews, as no new themes emerged. The first author then conducted two additional interviews in June 2021 to rule out the possibility of new information coming to light (Bryman, 2012). This was not the case, after which the researchers decided to finish data collection. The interviews were conducted in Dutch by telephone or via a video chatting platform. The length of the interviews varied from 40 to 70 min. All interviews were recorded and transcribed verbatim. Confidentiality was preserved by removing identifying details.

**TABLE 1:** Overview of participant characteristics

Respondent number	Gender	Age range	Team size	Type of hospital	Clinical unit	Interview conducted by telephone or video platform
1	Female	25–30	70	Academic hospital	Emergency department	Zoom
2	Female	18–24	60	Academic hospital	Pulmonary medicine	Zoom
3	Female	18–24	50	Regional hospital	Oncology (internal medicine)	Zoom
4	Female	25–30	35	Regional hospital	Emergency department	Telephone
5	Female	18–24	N/A	Academic hospital	Flex (at time of study: internal medicine and pulmonary medicine)	Zoom
6	Female	18–24	20	Regional hospital	Orthopaedics	Zoom
7	Female	18–24	100	Academic hospital	Obstetrics and gynaecology	Zoom
8	Female	18–24	100	Regional hospital	Surgery	Zoom
9	Female	18–24	15	Academic hospital	Transplantation and intestinal surgery	Zoom
10	Female	18–24	40	Regional hospital	Children's ward	Zoom
11	Female	18–24	40	Regional hospital	Children's ward	Zoom
12	Female	25–30	50	Academic hospital	Haematology	Zoom
13	Male	25–30	75	Academic hospital	Anaesthesiology	Zoom
14	Female	18–24	50	Academic hospital	Pulmonary medicine	Zoom
15	Female	18–24	30	Regional hospital	Pulmonary medicine	Zoom
16	Female	25–30	50	Regional hospital	Emergency department	Zoom
17	Female	25–30	30	Academic hospital	Pulmonary medicine	Zoom

### 2.4 Data analysis

Data analysis was conducted by using the constant comparative method (Bryman, 2012) and took place in three phases. First, both researchers read the transcripts several times in order to become familiar with the data. In the second phase, both researchers independently coded a selection of three interviews line‐by‐line. Coding was done using Atlas.ti (Atlas.ti GmbH, Berlin, Germany, Version 8.1). Exemplary codes were ‘Perceived lack of knowledge’, ‘Reflection sessions with co‐workers’, ‘Receiving help from a mentor’ and ‘Difficult relationship with physicians’. The researchers met and discussed the emerging findings and their initial codes with each other. This resulted in an initial coding scheme, which was then applied to subsequent transcripts and was further refined as new codes emerged. In doing so, we became aware that how NGRNs experienced their work and their relationships at work affected their voice behaviour. The third and final stage involved a further analysis of the coded dataset, oriented specifically towards the factors respondents perceived to be affecting their voice behaviour. Specifically, we organized the codes into descriptively labelled themes (e.g. ‘Being encouraged to speak up’ and ‘Prior voice experiences’). We discovered that seven factors affected NGRNs' voice behaviour, which we then grouped in four overarching themes.

### 2.5 Qualitative rigour

We used a variety of strategies to enhance qualitative rigor (Bryman, 2012). To establish credibility, both researchers analysed the data. Moreover, we used member‐checking: The emergent findings and interpretations were shared with two respondents to check for accuracy and resonance with their experiences. To establish dependability, we provide detailed descriptions of the data collection and analysis and offer quotes, which allows readers to verify our interpretations. Finally, to establish trustworthiness, we worked with reflexive journals and had regular meetings to discuss the (emergent) findings.

In preparing this manuscript, the Consolidated Criteria for Reporting Qualitative Studies guidelines (COREQ) were followed (Tong et al., 2007) (see Appendix A).

## 3 RESULTS

Our analysis of the data showed that whether NGRNs spoke up depended on (1) their levels of self‐confidence, (2) whether they felt encouraged and welcome to speak up, (3) their relationship with the voice target and (4) the content of their voice message. We will discuss this in more detail below. Quotes that illustrate our findings can be found in Table 2.

**TABLE 2:** Exemplar quotes

Factors influencing newly graduated registered nurses' voice behaviour		
NGRNs' levels of self‐confidence	Overcoming insecurities	‘I did not really know what I could expect. Everything was so new, so you do not speak up that fast’ (Respondent 2) ‘You have to process so much new info, have to perform different types of tasks. It was really a lot in the beginning and it has made me quite insecure. Instead of getting involved in discussions right away, I prefer to wait. I need to feel a bit more at ease and secure before I come more vocal’ (Respondent 6) ‘What frightened me a bit was that from one day to the next I was graduated and on my own. I was responsible for my patients. That was actually quite scary’ (Respondent 8)
	Support from a mentor	‘There needs to be a good induction programme, so you get to know your colleagues and the department. And you need to be paired with someone that you can discuss your issues with and who can help you in case you are struggling with something. Like a mentor’ (Respondent 14) ‘My mentor is really there for me. I have the feeling that I can discuss everything with her’ (Respondent 15)
	Gaining knowledge and experience	‘You go to school for four years and complete some internships, but your practical experience is basically zero. If you are a fulltime student, you just lack the skills. Yes, you have some knowledge, but it is just not enough to act as a fully qualified professional’ (Respondent 1) ‘You notice that once you gain knowledge and practical experience, you also become more daring to speak up’ (Respondent 11) I have learned a lot during my internships. And because of that, I feel equipped to think along. For instance, you learn to recognize certain disease states more quickly. So, you can think along, like: ‘Hey, maybe this patient has this disease, so we can give him this medication?’ (Respondent 13)
Feeling encouraged and welcome to speak up	Being stimulated to speak up	‘Colleagues also encourage me to speak up. They keep telling me: “We are the ones who actually interact with our patients, we know what is going on. Doctors need our input, so speak up!”’ (Respondent 7) ‘I was literally drilled to speak up, in the sense that my supervisors just kept encouraging me to speak up: “Does a doctor not disinfect his hands? Speak up about this! Speak up if you see something and do not be afraid to do so”’ (Respondent 12)
	Prior voice experiences	‘Last year, a lot of children were infected with the RS‐virus. During that time, I once experienced an acute situation. One of the children was not breathing, and because I was relatively new, I did not know how the equipment worked, so I could not help my colleagues. Afterwards, I sent an email to my supervisor and asked her for more training. She immediately undertook action and one week later the training took place (…) I had the feeling that my concerns were actually heard and that I was actually taken seriously, which has made me more vocal’ (Respondent 10) ‘If you mention something to the physician and you get a positive response, that gives you some satisfaction. Otherwise, you have the feeling you are not taken seriously, and that lowers your willingness to speak up’ (Respondent 14) ‘I notice that I enter into discussions more quickly. When I just came here, I was quite hesitant to do so, but I have spoken up a couple of times now. People actually listened to what I had to say and responded very positively, so now I am more daring to speak up’ (Respondent 17) ‘I once reported an incident to the head nurse, and then this particular colleague that was involved in this incident came to me, very angry, like: “Who do you think you are for reporting this?!” That really deterred me. I now think twice before speaking up and sharing my concerns’ (Respondent 2) Respondent: ‘When I worked in the cardiology department, one of the nurses made nasty comments. (…) The whole time, she was like: “This is wrong, and that is not okay.”’ Interviewer: ‘How did it make you feel?’ Respondent: ‘Well, you just become afraid to speak up’ (Respondent 11)
Relationship with the voice target	‘My team consists of a lot of novice registered nurses, who are all new to this department and hospital. We all need to learn, which creates mutual understanding. I feel really at ease in my interaction with them’ (Respondent 3) Despite me being here only for a short amount of time, I feel more and more at ease in my interaction with colleagues, because they behave respectfully and are friendly. Because of that, I dare to speak up (Respondent 12) ‘I worked at the surgery department and I actually felt like a slave. I just had to do what the doctor told me and often, they did not even know my name. It is so much different here. They even ask me like: “Hey, how was your holiday?” It is much more personal and that makes it a lot easier to speak up to physicians’ (Respondent 10) ‘In the hospital where I used to work, there existed a large hierarchy. I did not dare to speak up to the cardiologists. Here, in this hospital, the cardiologists are really friendly and approachable, they actually ask me how my weekend was. I now experience a lower threshold to make myself heard’ (Respondent 16)	
Content of the voice message	‘Well, when you think someone performed a care task in the “wrong way”, it is quite easy to speak up about that, because you can just refer to the protocol and can explain why you think it should be done differently. But sometimes you see things happening and you think: this is not okay. But then it is more personal, my own opinion. And then I have a hard time speaking up, because this other person may think it is a personal attack’ (Respondent 1) ‘When you have something that can back up your claim, it is easier to speak up. For instance, we work with a scoring system. When someone has a high heart rate or a low blood pressure, a patient gets a certain number of points. Once that patient reaches a certain score, you call the physician’ (Respondent 7) ‘Speaking up about things that a colleague did wrong, is super difficult—especially when it concerns the interaction with a patient’ (Respondent 9)	

### 3.1 NGRNs' levels of self‐confidence

#### 3.1.1 Overcoming insecurities

Most NGRNs experienced feelings of insecurity at the start of their contracts that refrained them from speaking up. Whereas during their internships they could rely on their supervisors, NGRNs now had final responsibility for one or more patients, which they found difficult. Moreover, as respondents started working at departments that were different from the ones in which they had completed their internships, they had to familiarize themselves with new patient populations, disease states and policies and protocols. For eight respondents, the hospital was even a new work environment, as they had completed their internships in nursing homes. These respondents shared that they had experienced a ‘transition shock’ ever since transitioning to the hospital, because of the more complex diseases they came across, the additional responsibilities that were part of their role and the different procedures and policies they had to work with. Respondents described that they had experienced this transition as tough and that they felt overwhelmed. This, in turn, had made them insecure. As such, rather than speaking up right away, respondents shared that they first had to adapt to their new work environment and become more confident before speaking up.

#### 3.1.2 Support from a mentor

Several respondents had been assigned a mentor, who acted as a point of reference and who provided the NGRNs with information and guidance during the first 3–5 months of their employment. The mentor was a more experienced colleague (>5 years of work experience) who knew the department well. Several respondents described their mentor as someone they could fall back on in case they were insecure or had questions. Consequently, NGRNs started to feel more confident in performing their jobs, as they could call their mentor in times of stress or uncertainty. As such, mentors helped the NGRNs overcome some of the initial insecurities they experienced, which helped NGRNs feel more at ease.

Moreover, although NGRNs initially were a bit hesitant to speak up, the good relationships most NGRNs had developed with their mentors ensured that they had a point of contact who they felt at ease to share their suggestions, recommendations and concerns with. One of the NGRNs, for instance, mentioned that during one of her first shifts, she had discovered that one of her patients had been given the wrong type of medication the night before. As she did not know the other colleagues in the department well enough, she decided to approach her mentor first and share this observation with her. The mentor then checked whether this patient had indeed been given the wrong type of medication, and, after realizing this was the case, the NGRN and her mentor together reported this incident. The respondent shared that this positive experience had made her more confident. As is discussed in more detail below, positive prior experiences with speaking up are an important facilitator of NGRNs' voice behaviour.

Moreover, mentors often acted as ‘linking persons’ between the NGRNs and other colleagues. That is, mentors introduced the NGRNs to other colleagues in the department, and, in doing so, facilitated communication. As is discussed further below, the development of relationships with colleagues also facilitated NGRNs' voice behaviour.

#### 3.1.3 Gaining knowledge and experience

NGRNs' knowledge base moreover affected their opportunities to speak up. Most respondents perceived that their training had provided them with basic nursing knowledge, but that deepening of this knowledge was needed in order to act as an autonomous professional. Because NGRNs did not possess all necessary knowledge right away, they indicated that they were not always able to identify problems, engage in discussions with other care personnel and come up with recommendations, for instance, regarding alternative treatment options. As such, NGRNs perceived that a lack of knowledge hindered their opportunities to engage in voice behaviour. Yet, as NGRNs gained knowledge and work experience, they noticed that they became more self‐confident, and, as a result, they felt more equipped to think along.

### 3.2 Feeling encouraged and welcome to speak up

#### 3.2.1 Being stimulated to speak up

NGRNs shared that receiving explicit invitations to speak up also enhanced their voice behaviour, as they felt encouraged and welcome to speak up. In several departments, initiatives, such as ‘reflection sessions’ and ‘care evaluation sessions’ were organized in which all personnel, including NGRNs, were explicitly invited to share their experiences, opinions, recommendations and concerns. In addition to these more formal initiatives, several respondents shared that also on the work floor itself, especially in the departments where acute care is being delivered, the importance of voice behaviour was emphasized on a day‐to‐day basis by colleagues and supervisors. Consequently, the respondents shared that they were oftentimes literally encouraged to speak up—also towards staff in higher hierarchical positions, which enhanced their voice behaviour.

#### 3.2.2 Prior voice experiences

Positive prior experiences with speaking up, that is, instances in which NGRNs had experienced that they were being listened to and that their voice was acted upon, also facilitated NGRN's voice behaviour. One NGRN, for instance, shared that during one of her shifts, a patient had received too much antibiotics. After signalling this to the head nurse, action was undertaken, and this particular NGRN received compliments from both colleagues and physicians. NGRNs derived satisfaction from these moments, perceived that their input actually mattered and felt that they were taken seriously. This, in turn, stimulated them to speak up more often.

Yet, other NGRNs shared more negative experiences. Respondents, for instance, had encountered that physicians were unwilling to listen to them because of their limited experience or ‘simply because they were “only” an NGRN’ (Respondent 8). Instances like these made respondents wonder whether they could actually make a difference by speaking up, which made them hesitant to do so.

### 3.3 Relationship with voice target

Whether NGRNs spoke up also depended on the relationship they had with the target of their voice message. One NGRN shared in this regard: ‘Whether I actually speak up, depends on who I have to speak up towards’ (Respondent 6). Most NGRNs shared that they had built relationships with other novice registered nurses in their teams that were characterized by mutual understanding and respect. Consequently, NGRNs often felt safe and at ease in their interaction with these nurses, which positively affected their voice behaviour.

Eight respondents shared that they were hesitant to speak up to older, more experienced nurses, as they perceived that the latter often acted pedantic and bossy towards the NGRNs. This became particularly apparent at moments when NGRNs proposed new ideas or offered suggestions for change. Six NGRNs had experienced that despite their good intentions, their input was met by deaf ears or was responded to defensively. While proposing a new, more efficient lifting technique to help patients out of bed, one of the NGRNs for example heard one of her more experienced co‐workers say: ‘Do not get involved in things that are none of your business’ (Respondent 17). Responses like these made NGRNs believe that speaking up to experienced nurses would not make a difference, which made them hesitant to do so.

Respondents also differed in the extent to which they were willing to speak up to physicians. Half of the respondents perceived the physicians in their departments to be friendly and approachable. What these respondents particularly liked was that physicians showed interest and asked how NGRNs were doing. Because of these friendly interactions, respondents felt at ease speaking up. However, not all respondents had such positive experiences. One nurse, for instance, shared how a physician snapped at her, whereas another respondent mentioned how she was ignored by physicians. This negatively affected their voice behaviour: ‘At one point, I experienced such a large hierarchy in my relationship with the cardiologists, that I did not even dare to call them anymore’ (Respondent 3).

### 3.4 Voice content

Finally, the content of the voice message affected NGRNs' voice behaviour. Specifically, respondents shared that they found it relatively easy to speak up to colleagues who did not adhere to safety protocols and regulations. Guidelines and protocols specified how optimal and safe care had to be delivered and how certain tasks had to be performed. NGRNs referred to these guidelines and protocols in order to back up their claims. One NGRN, for instance, had discovered that one of her colleagues had changed a catheter in a rather old‐fashioned way and not in accordance with current guidelines. The NGRN spoke up about this, and, in doing so, referred to the updated guidelines, to illustrate her point.

However, NGRNs were hesitant to speak up about matters for which no clear guidelines and regulations existed and which contained a value judgement. For instance, several NGRNs perceived that colleagues sometimes interacted with patients in an unpleasant manner. However, NGRNs believed this to be a personal matter, as nurses all had developed their own ways of interacting with patients. Consequently, most NGRNs were hesitant to speak up about matters like these, especially towards colleagues higher in status and hierarchy, as the latter could interpret this voice behaviour as a ‘personal attack’ or ‘negative feedback’.

## 4 DISCUSSION

**FIGURE 1:**
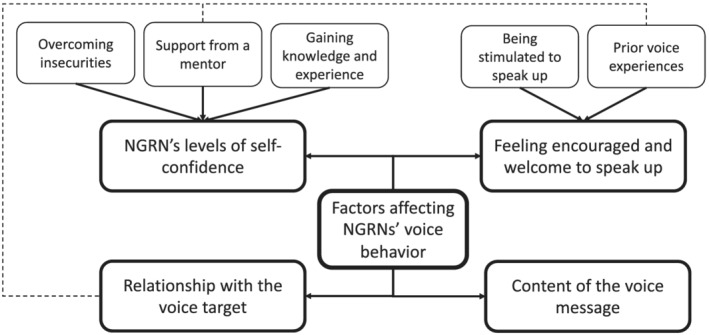
Overview of factors affecting newly graduated registered nurses' voice behaviour

Nowadays, the importance of nurses' voice behaviour is widely recognized (Levine et al., 2020; Morrow et al., 2016). However, the factors that influence NGRNs' voice behaviour remain largely unexplored. The present study aimed to fill this gap. In total, seven factors emerged from our data, which were grouped in four overarching themes. These are illustrated in Figure 1. Whether NGRNs speak up depends on (1) their levels of self‐confidence, (2) whether they feel encouraged and welcome to speak up, (3) their relationship with the voice target and (4) the content of their voice message. In what follows, we discuss our main findings in light of the existing literature.

A first insight that emerges from our research is the importance of time. That is, immediately after graduating, NGRNs may not feel at ease with speaking up, nor may be able to do so in the first place. In line with existing research, our study shows that NGRNs may face significant challenges as they transition to professional nursing practice (Lyman et al., 2020; Rush et al., 2019). The uncertainties that come with this transition make NGRNs insecure, which, in turn, and as we have shown, may prevent them from speaking up. In addition, NGRNs may not always be able to speak up due to a (perceived) lack of knowledge. In particular, a lack of practice experience and more specialized types of knowledge prevents NGRNs from engaging in discussions. As NGRNs grow into their roles, such hindrances can be overcome, and, over time, they may become more vocal.

Second, our research adds further credence to the relational nature of nurses' voice behaviour (Kee et al., 2021; Weiss et al., 2018). Our study is in line with existing scholarly work that has demonstrated that organizational hierarchies and status differences between members of different occupational groups are one of the main barriers to nurses' voice behaviour (Hanson et al., 2020; Morrow et al., 2016). However, in contrast to most previous studies, a few NGRNs also reported that their voice behaviour was hindered because of the behaviour executed by members of their own profession. Johnstone and Kanitsaki (2008) already pointed out that staff attitudes may pose a significant barrier to graduate nurse integration into hospital organizational and system processes. We add to this that such dynamics among occupational members may also affect voice behaviour.

Finally, our study adds to a growing understanding of how the content of nurses' voice messages may affect their willingness to speak up (Martinez et al., 2015). Existing research has shown that speaking up about patient safety concerns is becoming more normalized, as this has been incorporated into safety curricula, whereas speaking up about unprofessional behaviour and organizational misconduct is still difficult for nurses, as this remains largely untaught (Clancy & Tornberg, 2007). The latter is largely in line with our findings. However, whereas existing research shows that nurses refrain from speaking up about unprofessional behaviour and related topics that contain a value judgement as they believe this to be more confrontational and less acceptable (Martinez et al., 2015), we show that nurses—in particular NGRNs—are hesitant to do so, as speaking up about these matters may be interpreted as a personal attack or negative feedback, which may jeopardize existing professional relationships.

### 4.1 Study limitations

An important limitation of this study is the number of NGRNs interviewed. In total, 17 NGRNs were interviewed for the current study. Even though data saturation was reached, there is still a possibility that other factors affect NGRNs' voice behaviour as well. Moreover, as convenience and snowball sampling methods were used, representativeness of the sample is not guaranteed (Bryman, 2012). To overcome these shortcomings, survey research could be conducted, in which a larger, representative sample of NGRNs are asked about their experiences with speaking up. In doing so, also investigator bias can be overcome, as all participants are provided with a standardized questionnaire (Bryman, 2012).

Moreover, the current research focused on NGRNs who worked in hospital settings. NGRNs who work in care homes or who provide care in the community may have different experiences with speaking up. Repeating the current investigation and including NGRNs with different backgrounds could further increase our understanding of factors that affect NGRNs' voice behaviour.

### 4.2 Implications

Voice behaviour in the workplace is crucial for patients, care organizations and nurses themselves. Our research poses several directions nurse managers can take to support NGRNs' voice behaviour. Our findings demonstrate that NGRNs become more willing to speak up once they build self‐confidence. An induction programme provides an opportunity to welcome NGRNs to their team and to clarify the requirements, duties and responsibilities of their role. This, in turn, can help NGRNs in overcoming the initial insecurities they experience at the start of their employment. Similarly, gaining knowledge also made NGRNs more self‐confident, which enhanced their voice behaviour. Nurse managers could therefore organize additional training sessions for NGRNs.

Developing a mentoring programme may be helpful as well. As already pointed out by Gan (2021), such mentorship ideally starts when nurses are still in nursing schools, as more experienced nurses can support nursing students, and, eventually NGRNs, as they transition into their new roles. Regardless the moment a mentor becomes involved, mentors can act as informal points of reference and can provide information and guidance. This, in turn, will make feel NGRNs more at ease at work, which increases the chances of them speaking up.

Moreover, as our study has shown, mentors are often the first persons NGRNs speak up towards. It is therefore important for mentors to react to NGRNs' voice behaviour in a positive, constructive way and, as such, to ensure that NGRNs have positive, initial voice experiences. After all, our study has shown that once NGRNs believe their voices are being heard and acted upon, their willingness to speak up increases. Nurse managers may therefore offer additional training to mentors, in which mentors are learned about the importance of positive responses to NGRNs' voice.

Yet, not everything is within the control of nurse managers in a specific unit. Specifically, our study has demonstrated that colleagues play a vital role as well. Colleagues could enhance NGRNs' voice behaviour by explicitly inviting NGRNs to speak up and by explaining the importance of voice behaviour for the delivery of high‐quality and safe patient care. Moreover, in this study, NGRNs were more willing to speak up when they had high‐quality relationships with their colleagues. As such, colleagues can spend time to get to know NGRNs, be supportive of NGRNs and can act respectfully towards them. Although great efforts are needed to implement these changes, the rewards are worth it: more satisfied staff, better functioning care organizations and better and safer patient care.

## CONFLICT OF INTEREST

The authors declare that there is no conflict of interest.

## ETHICAL APPROVAL

Ethical approval was obtained from the Ethics Review Committee of the Faculty of Social Sciences, Vrije Universiteit Amsterdam. The authors completed an ethics self‐check. As the research complied with the ethical guidelines of the university, no further ethics review was deemed necessary.

## Data Availability

Data are available upon reasonable request.

## APPENDIX A COREQ 32‐ITEM CHECKLIST

**Table:**

No.	Item	Question	Answer
Domain 1: Research team and reflexivity			
*Personal characteristics*			
1	Interviewer/facilitator	Which author/s conducted the interview or focus group?	Both researchers conducted the interviews
2	Credentials	What were the researcher's credentials?	KK: MSc; DJ: MSc
3	Occupation	What was their occupation at the time of the study?	KK: PhD candidate; DJ: Junior researcher
4	Gender	Was the researcher male or female?	Both researchers are female
5	Experience and training	What experience or training did the researchers have?	Both researchers received training in qualitative research methodology and had experience with conducting interviews
	*Relationship with participants*		
6	Relationship established	Was a relationship established prior to study commencement?	Each interview started with an ‘introduction phase’, in which both researcher and respondent introduced themselves and respondents were asked about their (work) day and how they were doing
7	Participant knowledge of the interviewer	What did the participants know about the researchers?	All participants were informed of the reasons for doing the study and goals of the study. Moreover, both researchers explained who they were
8	Interviewer characteristics	What characteristics were reported about the interviewer?	At the start of each interview, the researcher briefly explained her background and interest in the research topic
Domain 2: Study design			
*Theoretical framework*			
9	Methodological orientation and theory	What methodological orientation was stated to underpin the study?	Qualitative descriptive design
10	Sampling	How were participants selected?	Convenience and snowball sampling
11	Method of approach	How were participants approached?	Telephone and email
12	Sample size	How many participants were in the study?	17
13	Non‐participation	How many people refused to participate or dropped out?	0
*Setting*			
14	Setting of the data collection	Where was the data collected?	One interview by telephone, 14 interviews via Zoom (during two interviews, two respondents were interviewed)—all respondents were at home at the time the interview was conducted
15	Presence of non‐participants	Was anyone else present besides the participants and researchers?	No
16	Description of sample	Description of sample	All respondents were newly graduated registered nurses with less than 2 years of work experience
*Data collection*			
17	Interview guide	Were questions, prompts, guides provided by the authors? Was it pilot tested?	The researchers together developed an interview guide that was based on prior interview guides developed by Garon (2012) and Schwappach and Gehring (2014). The authors asked open‐ended questions and encouraged the respondents to provide examples
18	Repeat interviews	Were repeat interviews carried out	No repeat interviews were carried out
19	Audio/visual recording	Did the researcher use audio or visual recording to collect the data?	All interviews were audio recorded
20	Field notes	Were field notes made during and/or after the interview?	Both researchers made field notes of the behaviours observed during the interviews and wrote down personal reflections after the interview had ended
21	Duration	What was the duration of the interviews?	40–70 min
22	Data saturation	Was data saturation discussed?	After 13 interviews, no new themes and dimensions emerged. By means of a member‐check, KK conducted two extra interviews, which again did not result in additional themes or dimensions
23	Transcripts returned	Were transcripts returned to participants for comment and/or correction?	The transcripts were not returned
Domain 3: Analysis and findings			
*Data analysis*			
24	Number of data coders	How many data coders coded the data?	Both researchers coded the data
25	Description of the coding tree	Did authors provide a description of the coding tree?	A description of the coding tree can be found in Section 2.4
26	Derivation of themes	Were themes identified in advance or derived from the data?	Themes were derived from the data
27	Software	What software; if applicable, was used to manage the data?	Atlas.ti
28	Participant checking	Did participants provide feedback on the findings?	Two participants received a draft version of the results, which they commented on
*Reporting*			
29	Quotations presented	Were participants' quotations presented to illustrate the themes/findings? Was each quotation identified?	Quotations to illustrate *all themes* have been provided, as well as the corresponding respondent numbers (Table 2)
30	Data and findings consistent	Was there consistency between the data presented and the findings?	There is consistency between the data presented and the findings
31	Clarity of major themes	Were major themes clearly presented in the findings?	Major themes are presented in Section 3 and presented in Table 2
32	Clarity of minor themes?	Is there a description of diverse cases or discussion of minor themes?	Diverse cases and minor themes are discussed in Section 3
